# The Other Side of the Question

**Published:** 1914-10

**Authors:** 


### The Other Side of the Question

While it is wise and proper to recognize clearly that the medical profession is overcrowded, it should also be recognized that the removal of competition is a delicate matter and that attempts in this direction should not pass the bounds of right and reason. We stand firmly against anything like compelling physicians already licensed to walk the plank, either because they have not anticipated requirements introduced at a later day or under any other pretext. We hold that, whatever the ratio of physicians to population, the individual youth has a right to choose his vocation and that neither educational requirements, tuition fees nor any other factor should be adjusted to a prohibitive scale beyond what is reasonable and proper irrespective of the law of supply and demand. And, for medical colleges, we hold that the same attitude should be taken. It may be desirable to have fewer colleges for economic reasons and better equipped colleges for scholastic reasons but we have no sympathy with the proposal to kill off redundant colleges or to raise the standards of maintenance to a level beyond the possibilities of all but a few favored by purely extrinsic aids not available to the majority.

